# Predictive value of LDL/HDL ratio in coronary atherosclerotic heart disease

**DOI:** 10.1186/s12872-022-02706-6

**Published:** 2022-06-17

**Authors:** Ting Sun, Mengyun Chen, Huanhuan Shen, PingYin, Li Fan, Xin Chen, Jun Wu, Zuojun Xu, Junfeng Zhang

**Affiliations:** 1 Department of Cardiology, Shanghai Ninth People’s Hospital Affiliated With Shanghai Jiao Tong University School of Medicine, Shanghai, 200025 People’s Republic of China; 2 Huamu Community Health Service Center, Pudong New Area, Shanghai, 201204 People’s Republic of China; 3 Community Health Service Center, Jiangwan Town, Hongkou District, Shanghai, People’s Republic of China; 4 Department of Laboratory Medicine, Shanghai General Hospital Jiading Branch, Shanghai, 201803 People’s Republic of China

**Keywords:** LDL/HDL ratio, Coronary atherosclerotic heart disease, Risks

## Abstract

**Background:**

Dyslipidemia is one of independent risk factors for coronary atherosclerotic heart disease (CAHD). We determined whether the LDL/HDL ratio is better than LDL-C or HDL-C alone in predicting the severity of CAHD.

**Methods:**

We performed a retrospective study of 1351 patients with myocardial ischemia who underwent coronary angiography between January 2018 and December 2019 in Shanghai Ninth People’s Hospital. Spearman correlation analysis, logistic regression model, Cox proportional hazards model and multicollinearity were used to evaluate LDL/HDL ratio for predicting CAHD severity compared to LDL-C or HDL-C alone.

**Results:**

Higher LDL/HDL ratio was seen in CAHD patients than controls (2.94 ± 1.06 vs 2.36 ± 0.78, *P* < 0.05). LDL/HDL ratio was significantly associated with the severity of coronary vascular stenosis. The area under the ROC curve of LDL-C, HDL-C, LDL/HDL ratio used to predict CAHD are 0.574 (95% CI 0.547–0.600, *P* < 0.001), 0.625 (95% CI 0.598–0.651, *P* < 0.001), 0.668 (95% CI 0.639–0.697, *P* = 0.000), respectively. The cut-off value of LDL/HDL ratio is 2.517, and the sensitivity and specificity are 65% and 61%, respectively. LDL/HDL ratio was related to the prevalence of CAHD and the odds ratio (OR) was 2.39 [95% confidence interval (CI) 1.698–2.593, *P* = 0.00] in multicollinearity regression model.

**Conclusion:**

LDL/HDL ratio may become a better predictor of CAHD severity, compared to LDL-C or HDL-C.

## Introduction

Coronary atherosclerotic heart disease (CAHD) is the main cause of morbidity and mortality in the world [1], especially in China. Early identification of CAHD can effectively reduce the burden of medicine and decrease the mortality of CAHD. Dyslipidemia is the main risk factor of CAHD, including high levels of low-density-lipoprotein cholesterol (LDL-C) and triglycerides (TG) [2], low levels of high-density-lipoprotein cholesterol (HDL-C). It has been proven that dyslipidemia is involved in both occurrence and development of arteriosclerosis, which increases the incidence and mortality of CAHD [3, 4]. Framingham study confirmed that low level of HDL-C increased the incidence of CAHD related events, and regarded as one of the important indicators for cardiovascular disease [5]. Small dense LDL-C particles, as the main carrier of cholesterol in plasma, is easy to penetrate through endothelium cell membrane when vascular endothelium is injured, which can cause plaque formation and inflammation cascade, and “initial effect” in the development of atherosclerosis [2, 6]. Increasing LDL-C can also directly lead to vascular endothelial injury, facilitating LDL-C penetration into the endothelium to form atherosclerosis plaque on the artery wall and thrombosis in CAHD [7–10]. While HDL-C can strengthen the surrounding tissue of the arterial wall, preventing cholesterol deposition in the arterial wall, and promoting the recovery of damaged endothelial membrane [11].On the other hand, low HDL-C level fails to eliminate cholesterol efficiently, that can lead to early onset of atherosclerosis.

In clinical practice, we frequently encountered some patients with normal ranges of LDL-C and HDL-C who are prone to CAHD, for whom new indicators are needed to develop. Dyslipidemia include high LDL-C, low HDL-C, and high triglyceride or combined, which puts forward the following questions on which type of dyslipidemia is more likely to reflect the coronary atherosclerosis severity. It inspires us to investigate whether the combination of LDL-C and HDL-C more accurately reflects dyslipidemia. Previous studies have reported that increased LDL/HDL is related to myocardial infarction [12, 13], but the relationship of LDL/HDL and the coronary atherosclerosis severity is not illustrated. In this study, we investigate the clinical characteristics and serum lipids assays of 1351 subjects and clarify whether the LDL/HDL ratio is a better predictor for CAHD severity, than LDL-C or HDL-C alone.

## Materials and methods

### Study population

A retrospective analysis is performed on 1351 consecutive patients presenting to Shanghai Ninth People’s Hospital with signs of myocardial ischemia between January 2018 and December 2019. All patients fulfill the following inclusion criteria and exclusion criteria. Inclusion criteria: (1) over 18 years old; (2) signs of myocardial ischemia; (3) undergoing invasive coronary angiography; Exclusion criteria: (1) pulmonary heart diseases; (2) acute myocardial infarction (AMI) and valvule heart disease; (3) congenital heart disease; (4) family hereditary hyperlipidemia; (5) malignant tumor and immune diseases; (6) severe hepatic or renal dysfunction; (7) incomplete clinical data. According to the World Health Organization guidelines [14] for the diagnosis of coronary heart disease [15], stenosis of any major coronary artery (left anterior descending branch, left circumflex branch, right coronary artery) or its main branches ≥ 50% is taken as the diagnostic criteria for CAHD. Primary hypertension is defined according to International Society of Hypertension, and Diabetes Mellitus is defined according to the World Health Organization essential diagnostic criteria [5, 16]. Demographic characteristics including age, sex, height, weight, CAHD family history, smoking status, diabetes mellitus and hypertension are recorded. The project is subject to the construction and application of biobank for coronary heart disease at Shanghai Ninth People's Hospital (YBKA201910).

### Laboratory determination and echocardiography

All subjects were investigated in the morning after an overnight fast on the third day of admission without acute ischemia. The serum lipids were enzymatically measured using the Hitachi 747 chemical analyzer (Hitachi, Tokyo, Japan) according to the manufacturer’s instructions. Serum creatinine (Cr), cardiac troponin I (cTn I), creatinine kinase, fasting blood glucose (FBG), glycated hemoglobin A1C (HbA1C), brain natriuretic peptide (BNP) and uric acid (UA) were measured using standard laboratory techniques. Transthoracic echocardiography (TTE) was performed in all patients by using an ultrasound device (ie33, Philips Medical System, Bothell, Washington, USA).

### Diagnostic coronary angiographic examinations and groups

Diagnostic coronary angiography (CAG) was performed on participants with unstable angina, chronic stable angina and atypical chest pain speculated as CAHD. Angiographies were performed using Judkins right and left catheters through the radial artery and a Germany’s Siemens Axiom Artis DFC ZEE floor mounted device. The quantification analysis of obstructions after catheter calibration was performed by moving the cursor from the proximal through the distal region of each vessel to determine the length and severity of the obstruction. Quantitative coronary angiography (QCA) was performed in biplane views. Patients were screened and classified into four groups: normal (one or more coronary stenosis < 10% in diameter), coronary artery atherosclerosis (one or more coronary stenosis 10–50% in diameter), and CAHD (one or more coronary stenosis ≥ 50% in diameter) in any coronary arteries, serious CAHD (one or more coronary stenosis  ≥70% in diameter) according to the International Statistical Classification of Diseases and Related Health Problems criteria (10th Revision) and the American Heart Association classification for cardiovascular diseases. The severity of coronary lesions was also evaluated by using the Gensini score system, based on the results of the CAG. In the Gensini score system, the lesions were classified as 0–25%, 26–50%, 51–75%, 76–90% according to the degree of angiographic stenosis, and were scored 1, 2, 4, 8, 16 and 32 points, respectively. Then the score was multiplied by the coefficient defined according to the localization of the lesion [17].

### Statistical analysis

Statistical analysis was performed using SPSS 26.0 software (SPSS Inc., Chicago, Illinois, USA). Data were expressed as percentages or the mean ± standard deviation. Two group comparisons were used with Student’s-tests (normally distributed) or nonparametric tests (non-normally distributed) for continuous variables and χ^2^ tests for categorical variables. Pearson correlation analysis was used to evaluate continuous variables with multiple groups. Univariate and multivariate logistic regression models were performed to distinguish all risk factors for CAHD. The Cox proportional hazards model and the multicollinearity regression model were used to illustrate the diagnostic power of LDL/HDL for coronary stenosis. The optimal cutoffs were derived from the receiver operating characteristic (ROC) curve by maximizing the sum of sensitivity and specificity. Two-sided *P* values < 0.05 were considered statistically significant.

## Results

### Subject characteristics

There are 1351 participants (696 males and 655 females; mean age of 66.51 ± 10.44 years, minimum 23, maximum 91) enrolled in this study, with no significant differences in education status; drinking status; medication history; arrhythmia; myocardial infarction history between CAHD and non-CAHD group (Table 1). The patients in CAHD group were older (*P* < 0.01) and more male, compared with those in non-CAHD group. CAHD group had more patients with CAHD family history; smoking; hypertension; diabetes mellitus; renal insufficiency; cerebral infarction; heart failure; angina pectoris than non-CAHD group (Table 1).

**Table 1:** Clinical Characteristics, cardiac function, complications and medication history of Studied Sample

Variable	Non-CAHD (n = 738)	CAHD (n = 613)	t/χ^2^ Value	*P* Value
Sex, Male (n%)	324 (44%)	372 (61%)	37.760	< 0.001
Age ≥ 65 y, (n%)	344 (47%)	350 (57%)	15.040	< 0.001
Education, (n%)			2.810	0.245
Illiteracy, (n%)	34 (5%)	40 (7%)		
Under high school (n%)	646 (88%)	518 (85%)		
Undergraduate course (n%)	55 (8%)	50 (8%)		
Smokers (n%)	198 (27%)	228 (37%)	17.025	< 0.001
Drinkers (n%)	151 (21%)	140 (23%)	1.197	0.274
Diabetes mellitus (n%)	137 (19%)	181 (30%)	22.710	< 0.001
Hypertension (n%)	332 (45%)	186 (30%)	29.720	< 0.001
LVEF, %	62.24 ± 4.19	60.32 ± 7.25	-3.946	< 0.001
CAHD family history (n%)	89 (12%)	143 (23%)	29.890	< 0.001
Angina pectoris (n%)	49 (6.7%)	76 (12.4%)	13.064	0.001
Old Myocardial Infarction (n%)	3 (0.4%)	8 (1.3%)	3.330	0.069
Heart failure (n%)	155 (21%)	202 (33%)	24.980	< 0.001
Arrhythmia (n%)	264 (36%)	204 (34%)	0.920	0.337
Cerebral infarction (n%)	108 (15%)	119 (19%)	5.430	0.020
Renal insufficiency (n%)	24 (3%)	37 (6%)	6.037	0.010
Antihypertensive drug (n%)	207 (28%)	170 (28%)	0.017	0.931
Hypolipidemic drug (n%)	144 (20%)	112 (18%)	0.330	0.561

*LVEF* left ventricular ejection fraction, *CAHD* coronary atherosclerotic heart disease

### The risk factors of CAHD

**Fig. 1:**
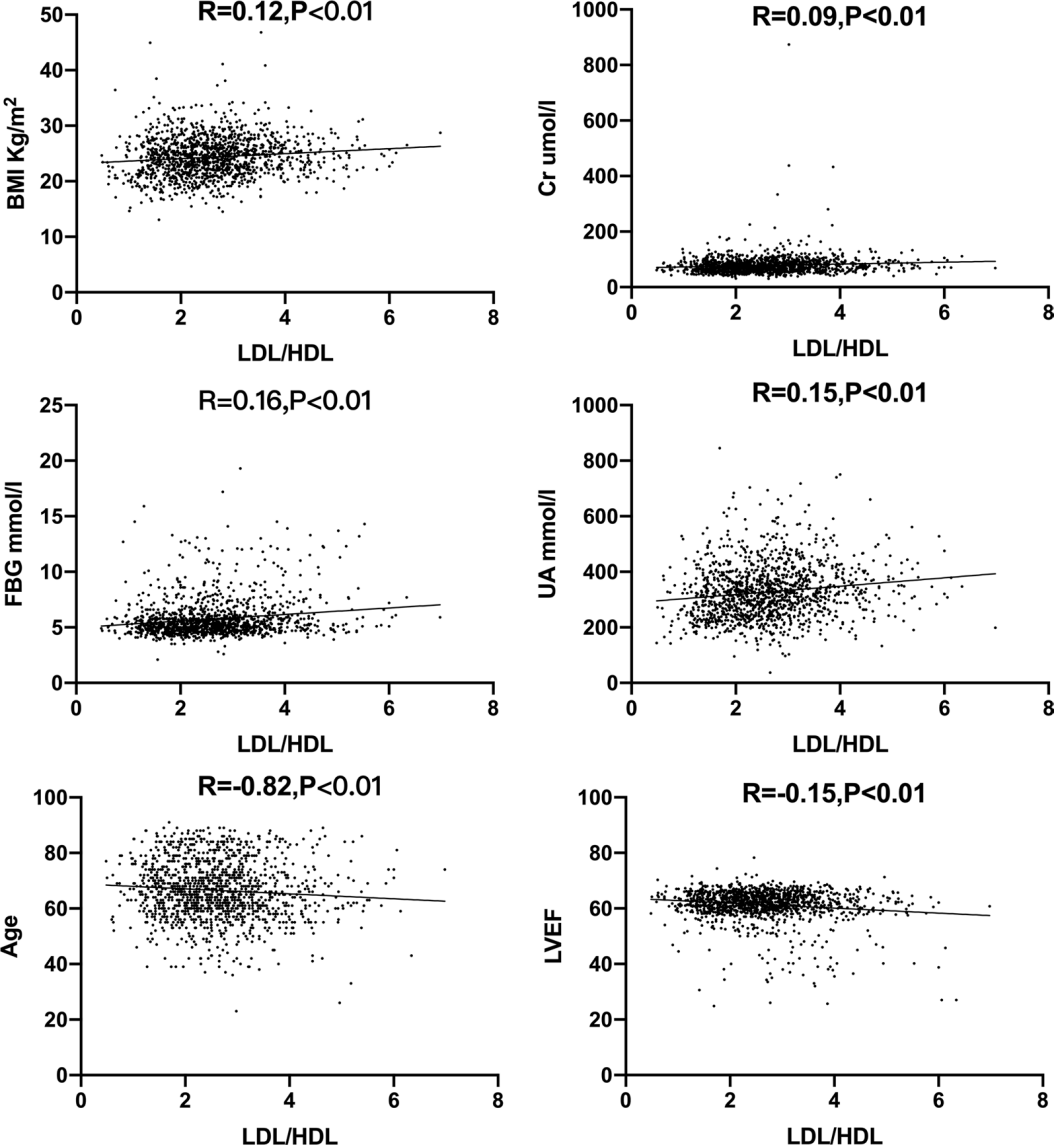
The correlation between LDL/HDL and age, BMI, Cr, UA, FBG and LVEF

Many factors in CAHD are shown in Tables 1 and 2. CAHD patients had significantly higher levels of serum Cr; FBG; HbA1C; BNP; cTnI; UA; LDL-C and lower LVEF; HDL-C than non-CAHD patients. LDL/HDL ratio was higher in CAHD group than that in non-CAHD group (2.94 ± 1.06U/L vs 2.36 ± 0.78U/L *P* < 0.01). As shown in Fig. 1, BMI, Cr, UA, GLU were positive, but age and LVEF were negative with LDL/HDL ratio. As shown in Table 3, there are significantly different in age; hypertension; BMI; LDL; HDL-C and coronary artery severity between DM group and non-DM group. In Fig. 1 there is a statistically significant association of coronary artery stenosis severity with age, sex, smoking, diabetes, and renal dysfunction. Alcohol consumption, history of arrhythmias, and history of other diseases were no difference in groups with different coronary artery stenosis severity.

**Table 2:** laboratory text index and Lipids levels of studied sample

Variable	Non-CAHD (n = 738)	CAHD(n = 613)	t/χ^2^ Value	*P* Value
LDL/HDL	2.361 ± 0.782	2.942 ± 1.063	− 10.670	0.000
Cr (μmol/L)	74.191 ± 35.152	83.151 ± 34.351	− 4.720	0.000
FBG (mmol/L)	5.571 ± 1.483	5.971 ± 2.013	− 4.180	0.000
HbA1C (%)	6.19 ± 0.89	6.53 ± 1.11	− 6.18	0.000
BNP (pg/mL)	74.31 ± 86.08	212.64 ± 454.34	− 7.210	0.000
CRP (mg/L)	3.222 ± 7.890	5.670 ± 8.622	− 3.366	0.001
HDL-C (mmol/L)	1.192 ± 0.351	1.041 ± 0.292	7.990	0.000
cTnI (ng/mL)	0.031 ± 0.151	0.802 ± 6.971	− 6.590	0.006
UA (μmol/L)	315.751 ± 92.321	341.723 ± 99.501	− 4.980	0.000
TC (mmol/L)	4.171 ± 0.952	4.281 ± 1.133	− 1.680	0.090
TG (mmol/L)	1.571 ± 1.334	1.691 ± 1.412	− 1.590	0.113
LDL-C (mmol/L)	2.672 ± 0.841	2.914 ± 0.951	− 4.670	0.000
Lp(a) (g/L)	0.142 ± 0.161	0.151 ± 0.182	− 1.360	0.175

Values are expressed as mean ± standard deviation; *LDL-C* low-density-lipoprotein cholesterol, *HDL-C* high-density-lipoprotein cholesterol, *LDL/HDL* the ratio of low-density-lipoprotein to high-density-lipoprotein, *Cr* serum creatinine, *FBG* fasting blood-glucose, *HbA1C* glycated hemoglobin A1C, *BNP* brain natriuretic peptide, *CRP* C-creactive protein, *cTnI* cardiac troponin I, *UA* uric acid, *TC* total cholesterol, *TG* serum triglyceride, *Lp*(*a*) lipoprotein(a)

**Table 3:** Clinical characteristics and lipids level in DM group

Categorical Variables	Diabetes mellitus		
	NO (n = 1038)	Yes (n = 318)	*P* value
Sex, male (n%)	532 (52%)	164 (52%)	0.982
Age			< 0.001
< 65 y	539 (52%)	122 (38%)	
≥ 65 y	499 (48%)	195 (61%)	
Smoker (n%)	710 (68%)	220 (69%)	0.793
Drinker (n%)	233 (22%)	58 (18%)	0.110
Hypertension (n%)	449 (43%)	69 (22%)	< 0.001
CAHD history (n%)	153 (14.8%)	79 (24.8%)	< 0.001
Heart failure history (n%)	250 (24%)	107 (34%)	0.001
Arrhythmia history (n%)	351 (34%)	117 (37%)	0.346
Cerebral infarction history (n%) (n%)	156 (15%)	71 (22%)	0.002
Renal insufficiency (n%)	40 (4%)	21 (6.6%)	0.040
Hypolipidemic drug (n%)	189 (18%)	68 (22%)	0.216
Coronary artery stenosis (n%)			< 0.001
< 10%	355 (34%)	49 (15%)	
10–50%	250 (24%)	88 (28%)	
50–70%	300 (29%)	102 (32%)	
≥ 70%	133 (13%)	79 (25%)	
BMI (kg/m^2^)			0.005
Underweight (BMI < 18.5)	45 (4%)	11 (4%)	
Normal (18.5 ≤ BMI < 23.9)	464 (45%)	113 (36%)	
Overweight (24 ≤ BMI < 27.9)	386 (37%)	130 (41%)	
Obese (BMI ≥ 28.0)	136 (13%)	62 (20%)	
Fasting blood glucose (mmol/L)	5.32 ± 1.05	7.17 ± 2.62	< 0.001
LDL/HDL	2.60 ± 0.95	2.69 ± 0.99	0.157
LDL-C			0.007
< 2.6 mmol/L	446 (43%)	164 (52%)	
≥ 2.6 mmol/L	592 (57%)	154 (48%)	
HDL-C			< 0.001
< 1 mmol/L	396 (38%)	165 (52%)	
≥ 1 mmol/L	642 (62%)	153 (48%)	
TC			0.251
< 5.2 mmol/L	867 (84%)	278 (87%)	
≥ 5.2 mmol/L	168 (16%)	40 (13%)	
TG			0.129
< 1.7 mmol/L	741 (71%)	210 (66%)	
≥ 1.7 mmol/L	296 (29%)	108 (34%)	
Ox-LDL (mmol/L)	0.312 ± 0.193	0.369 ± 0.443	0.027
HbA1C			< 0.001
≤ 6.5%	93.9%	31.4%	
> 6.5%	6.1%	68.6%	
Cr			< 0.001
< 97 μmol/L	912 (88%)	252 (79%)	
≥ 97 μmol/L	126 (12%)	66 (21%)	

Values are expressed as mean ± standard deviation or percentage

*BMI* body mass index, *LDL-C* low-density-lipoprotein cholesterol, *HDL-C* high-density-lipoprotein cholesterol, *LDL/HDL* the ratio of low-density-lipoprotein to high-density-lipoprotein, *TC* total cholesterol, *TG* triglyceride, *Cr* serum creatinine, *CAHD* coronary atherosclerotic heart disease, *DM* diabetes mellitus

### LDL/HDL ratio and CAHD severity

Table 4 show clinical characteristics and lipids levels in different groups according to atherosclerotic lesion severity. LDL/HDL ratio increased with the degree of coronary artery stenosis increased and with Gensini scores increased (*P* < 0.01). Patients with a lower HDL-C (58.5% vs 41.5% *P* < 0.01) and higher LDL-C (59.7% vs 40.3%, *P* < 0.01) demonstrate more severe coronary artery severity than other groups. Using categorical variables, the patients with high triglycerides in severe coronary atherosclerotic groups was more than that in mild coronary atherosclerotic groups. But no difference of total cholesterol and Lp(a) are found among different coronary artery severity groups.

**Table 4:** Clinical characteristics and lipids levels of the study sample according to the degree of coronary artery stenosis and the Gensini scores system

Categorical Variables	Coronary artery stenosis					Gensini scores				
	< 10% (n = 404)	10–50% (n = 337)	50–70% (= 402)	≥ 70% (n = 212)	*P* value	0–18 (n = 403)	19–39 (n = 367)	40–58 (= 385)	59- (n = 196)	*P* value
Sex, M (n%)	165 (41%)	159 (47%)	236 (59%)	136 (64%)	< 0.001	168 (42%)	174 (47%)	227 (59%)	128 (65%)	< 0.001
Age					< 0.001					< 0.001
< 65 y	245 (61%)	152 (45%)	177 (44%)	87 (41%)		242 (60%)	168 (46%)	169 (44%)	78 (40%)	
≥ 65 y	159 (39%)	185 (55%)	225 (56%)	125 (59%)		161 (40%)	198 (54%)	216 (56%)	118 (60%)	
Smoker (n%)	100 (25%)	98 (29%)	153 (38%)	75 (35%)	< 0.001	101 (25%)	106 (29%)	145 (38%)	74 (38%)	< 0.001
Drinker (n%)	70 (17%)	81 (24%)	92 (23%)	48 (23%)	0.110	71 (18%)	84 (23%)	89 (23%)	46 (24%)	0.167
DM (n%)	49 (12%)	88 (26%)	102 (25%)	79 (37%)	< 0.001	51 (13%)	95 (26%)	94 (24%)	78 (40%)	< 0.001
HTN (n%)	208 (52%)	124 (37%)	119 (30%)	67 (32%)	< 0.001	199 (49%)	234 (64%)	267 (69%)	139 (71%)	< 0.001
AH (n%)	139 (35%)	125 (37%)	134 (33%)	70 (33%)	0.714	139 (35%)	136 (37%)	130 (34%)	63 (32%)	0.714
HDU (n%)	76 (20%)	66 (20%)	70 (18%)	42 (20%)	0.828	383 (95%)	336 (92%)	300 (78%)	152 (78%)	0.620
BMI (kg/m^2^)					< 0.001					< 0.036
< 18.5	19 (5%)	9(3%)	16(4%)	12(6%)		16(4%)	9(3%)	10(2.6%)	10(6%)	
18.5–23.9	18 (45%)	147 (44%)	17 (43%)	80 (39%)		183 (45%)	162 (44%)	167 (43%)	77 (39%)	
24.0–27.9	14 (35%)	12 (36%)	16 (41%)	90 (44%)		142 (35%)	132 (36%)	164 (43%)	87 (44%)	
≥ 28.0	64 (19%)	59 (18%)	51 (13%)	24 (12%)		62 (15%)	63 (17%)	44 (11%)	22 (11%)	
LDL/HDL	2.49 ± 0.83	2.20 ± 0.69	2.84 ± 1.02	3.13 ± 1.11	< 0.001	2.48 ± 0.68	2.24 ± 0.73	2.90 ± 1.02	3.08 ± 1.13	< 0.001
LDL-C					< 0.001					< 0.001
< 2.6 mmol/L	173 (43%)	191 (57%)	162 (40%)	84 (40%)		176 (44%)	200 (54%)	147 (38%)	84 (43%)	
≥ 2.6 mmol/L	23 (57%)	147 (44%)	240 (60%)	128 (60%)		227 (56%)	167 (45%)	238 (62%)	112 (57%)	
HDL-C					< 0.001					< 0.001
≤ 1 mmol/L	142 (35%)	102 (30%)	193 (48%)	124 (59%)		145 (36%)	111 (30%)	190 (49%)	114 (58%)	
> 1 mmol/L	262 (65%)	236 (70%)	209 (52%)	88 (42%)		258 (64%)	256 (70%)	195 (51%)	82 (42%)	
TC					0.182					0.205
< 5.2 mmol/L	340 (84%)	297 (88%)	336 (84%)	172 (81%)		344 (85%)	321 (87%)	317 (82%)	162 (83%)	
≥ 5.2 mmol/L	63 (16%)	41 (12%)	64 (16%)	40 (19%)		59 (15%)	46 (13%)	68 (18%)	34 (17%)	
TG					0.009					0.007
< 1.7 mmol/L	298 (74%)	250 (74%)	268 (67%)	135 (64%)		298 (74%)	270 (74%)	251 (65%)	127 (65%)	
≥ 1.7 mmol/L	10 (26%)	88 (26%)	134 (33%)	77 (36%)		104 (26%)	97 (26%)	134 (35%)	69 (35%)	
Cr					< 0.001					< 0.001
< 97 μmol/L	368 (91%)	306 (91%)	333 (83%)	157 (74%)		363 (90%)	332 (91%)	324 (84%)	140 (71%)	
≥ 97 μmol/L	36 (19%)	32 (10%)	69 (17%)	55 (29%)		40 (10%)	35 (9%)	61 (16%)	56 (29%)	

Values are expressed as mean ± standard deviation

*M* male, *DM* diabetes mellitus, *HTN* hypertension, *AH* arrhythmia history, *HDU* hypolipidemic drug use, *LDL-C* low-density-lipoprotein cholesterol, *HDL-C* high-density-lipoprotein cholesterol, *LDL/HDL* the ratio of low-density-lipoprotein to high-density-lipoprotein. *BMI* body mass index, *TC* total cholesterol, *TG* serum triglyceride, *Cr* serum creatinine

### ROC curve analysis for LDL-C, HDL-C, LDL/HDL ratio and Predictive value for the risk of CAHD

**Fig. 2:**
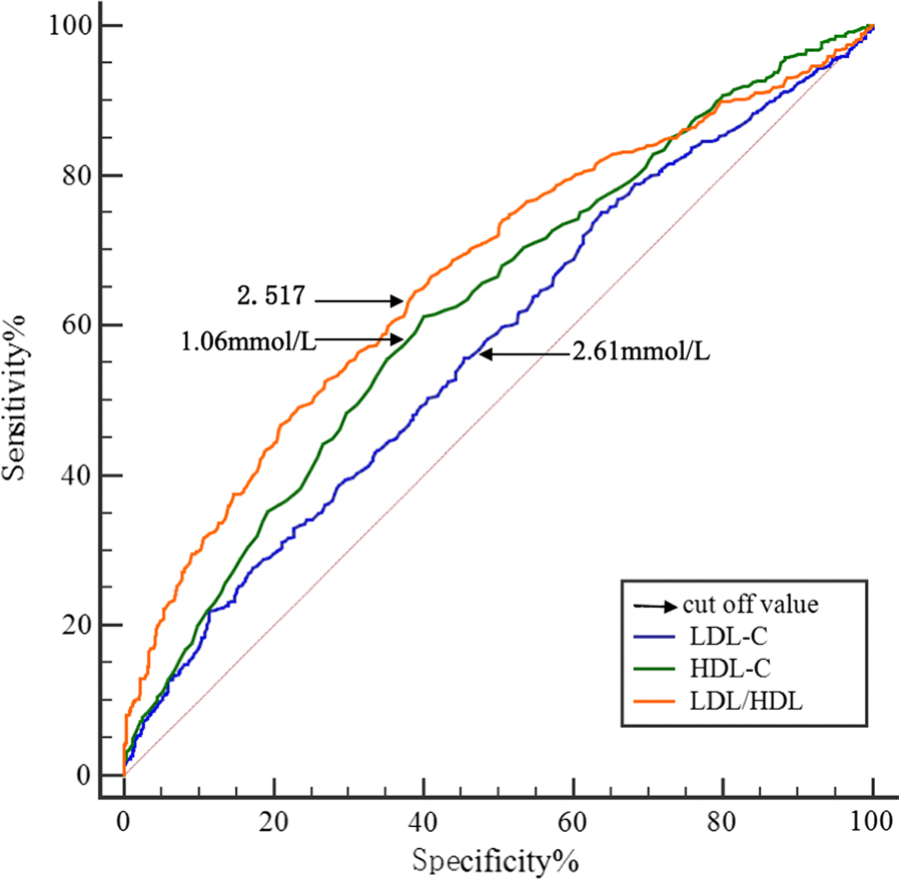
ROC analysis of LDL-C, HDL-C and LDL/HDL

The results of ROC analysis of LDL-C, HDL-C, LDL/HDL ratio are shown in Fig. 2 and Table 5. The area under the ROC curve (AUC) of LDL-C, HDL-C, LDL/HDL ratio for predicting the risk of CAHD were 0.574, 0.625, 0.668 (*P* < 0.001), respectively. The optimal cut-off value for LDL/HDL ratio was 2.517, and the sensitivity and specificity were 64.5% and 61.3%, respectively. As shown in Fig. 2, we compared the ROC curve among LDL-C, HDL-C and LDL/HDL ratio, and found LDL/HDL ratio is superior to LDL-C or HDL-C in predicting the coronary atherosclerotic severity.

**Table 5:** Comparative analysis of ROC curve for various cut-off levels of LDL-C,HDL-C, LDL/HDL

Variables	AUC/D-AUC (95%CI)	SE	Z	Sensitivity	Specificity	Cut-off value	*P* Value
LDL-C	0.574 (0.547–0.600)	0.016	4.714	75%	36%	2.32 mmol/L	0.000
HDL-C	0.625 (0.598–0.651)	0.015	8.233	61%	60%	1.06 mmol/L	0.000
LDL/HDL	0.668 (0.639–0.697)	0.015	11.327	65%	61%	2.517	0.000
LDL-C vs HDL-C	0.051 (0.003–0.099)	0.025	2.089	–	–	–	0.037
LDL-C vs LDL/HDL	0.095 (0.071–0.119)	0.012	7.723	–	–	–	0.000
HDL-C vs LDL/HDL	0.043 (0.012–0.074)	0.016	2.737	–	–	–	0.000

*ROC* receiver operating characteristic, *AUC* area under curve, *D-AUC* differential area under curve. Values are expressed as mean ± standard deviation or percentage. *LDL-C* low-density-lipoprotein cholesterol, *HDL-C* high-density-lipoprotein cholesterol, *LDL/HDL* the ratio of low-density-lipoprotein to high-density-lipoprotein

We divided the participants into two groups according to the cut-off value of LDL/HDL, and analyzed the incidence of CAHD and related factors. There was more incidences of CAHD and renal insufficiency, higher BMI, Creatinine, UA, more males and smokers in the LDL/HDL over 2.517 group than in the LDL/HDL under 2.517 group (Tables 6 and 7). More individuals with severe coronary artery stenosis were seen in LDL/HDL over 2.57 group. There was no significant difference in age, drinker, diabetes, BNP, LVEF and other history between the two groups.

**Table 6:** Risk factors predicted by the cut-off value of LDL/HDL in the study population

Categorical Variables	LDL/HDL		
	< 2.5173 (n = 673)	≥ 2.5173 (n = 683)	*P* value
Age			0.137
< 65 y	312 (46%)	349 (51%)	
≥ 65 y	360 (54%)	334 (49%)	
Sex			0.000
Male	306 (46%)	390 (57%)	
Female	367 (55%)	293 (43%)	
Smoker			0.001
Yes	183 (27%)	243 (36%)	
No	490 (73%)	440 (64%)	
Hypertension			0.003
Yes	284 (42%)	234 (34%)	
No	389 (58%)	449 (66%)	
DM			0.316
Yes	150 (22%)	168 (25%)	
No	523 (78%)	515(75%)	
BMI (kg/m^2^)			0.002
Underweight (BMI < 18.5)	32 (5%)	24 (4%)	
Normal (18.5 ≤ BMI < 23.9)	32 (48%)	25 (38%)	
Overweight (24 ≤ BMI < 27.9)	23 (35%)	28 (41%)	
Obese (BMI ≥ 28.0)	87 (13%)	11 (16%)	
Cr			0.000
< 97 μmol/L	602 (90%)	562 (82%)	
≥ 97 μmol/L	71 (11%)	121 (18%)	
CAS			0.000
< 10%	213 (32%)	191 (28%)	
10–50%	242 (36%)	96 (14%)	
50–70%	153 (23%)	338 (50%)	
≥ 70%	65 (10%)	147 (22%)	

Values are presented as number percentage

*LDL/HDL* the ratio of low-density-lipoprotein to high-density-lipoprotein, *DM* diabetes mellitus, *BMI* body mass index, *Cr* serum creatinine, *CAS* coronary artery stenosis

**Table 7:** Risk factors predicted by the cut-off value of LDL/HDL in the study population

Variable	LDL/HDL < 2.517 (n = 673)	LDL/HDL≥ 2.517 (n = 683)	*t/χ*^*2*^ Value	*P* Value
Sex (M, n%)	306 (46%)	390 (57%)	17.710	< 0.001
Age (year)	67.032 ± 10.203	66.001 ± 10.653	1.820	0.069
Smokers (n%)	183 (27%)	243 (36%)	11.070	0.001
Drinkers (n%)	136 (20%)	155 (23%)	1.243	0.265
DM (n%)	150 (22%)	168 (25%)	1.007	0.316
Hypertension (n%)	284 (42%)	234 (34%)	9.049	0.003
LVEF (%)	62.001 ± 4.623	60.752 ± 6.802	3.980	0.081
CAHD (n%)	218 (32%)	396 (58%)	12.080	0.001
HF history (n%)	164 (24%)	193 (28%)	2.644	0.104
ARR history (n%)	427 (64%)	460 (67%)	2.282	0.131
BMI (kg/m^2^)	23.943 ± 3.871	24.651 ± 3.703	− 3.490	0.001
LDL-C (mmol/L)	2.281 ± 0.682	3.271 ± 0.814	− 24.410	< 0.001
HDL-C (mmol/L)	1.252 ± 0.372	0.991 ± 0.231	15.580	< 0.001
TC (mmol/L)	3.852 ± 0.872	4.591 ± 1.053	-14.130	< 0.001
TG (mmol/L)	1.433 ± 1.311	1.823 ± 1.402	− 5.330	< 0.001
Lp(a) (g/L)	0.141 ± 0.181	0.141 ± 0.171	− 0.180	0.859
FBG (mmol/L)	5.571 ± 1.532	5.932 ± 1.933	− 3.850	< 0.001
HbA1c (%)	6.332 ± 2.503	6.463 ± 1.232	− 1.100	0.271
Cr (μmol/L)	74.123 ± 21.161	82.301 ± 44.361	− 4.340	< 0.001
UA (μmol/L)	315.002 ± 91.453	339.831 ± 99.723	− 4.780	< 0.001
CTnI (ng/mL)	0.221 ± 3.913	0.532 ± 5.372	− 1.190	0.467
BNP (pg/mL)	123.781 ± 282.084	149.941 ± 352.532	− 1.510	0.257

*BMI* body mass index, *HbA1C* glycoated hemoglobin A1C, *UA* uric acid, *TG* serum triglyceride, *Lp*(*a*) lipoprotein(a), *Cr* serum creatinine, *LVEF* left ventricular ejection fraction, *BNP* brain natriuretice peptide, *cTnI* cardiac troponin I, *FBG* fasting blood-glucose, *TC* total cholesterol, *HDL-C* high-density-lipoprotein cholesterol, *LDL-C* low-density-lipoprotein cholesterol, *LDL/HDL* the ratio of low-density-lipoprotein to high-density-lipoprotein, *M* male, *CAHD* coronary atherosclerotic heart disease, *DM* diabetes mellitus, *ARR history* arrhythmia history, *HF* heart failure

### Predictive value of LDL/HDL ratio for CAHD

The odds ratio (OR) and 95% confidence interval (95% CI) of the CAHD risk factors are shown in Table 8. Both in univariate and multivariate analyses, smoker, sex, age, hypertension, and DM were statistically associated with the prevalence of CAHD. Other factors, such as TC, BMI, hypolipidemic treatment, showed no predictive value for CAHD. These data suggest that older age, smoker, male, DM and hypertension are the risk factors of CAHD. HDL-C and LDL-C were also connected to the prevalence of CAHD in univariate analysis but not in multiple analyses. While LDL/HDL ratio was significantly associated with CAHD in both univariate and multivariate logistic regression analyses, as shown in Table 8. Therefore, LDL/HDL is a better risk predictor for CAHD than LDL-C or HDL-C alone. The standardized coefficient of each independent variable was showed in Table 9. Variance inflation factor (VIF) is near to 1 in multicollinearity regression model. Each independent variable can be used most effectively to predict CAHD and there is no collinearity between age, sex, hypertension, diabetes, smoking and LDL/HDL ratio.

**Table 8:** Univariate and multivariate logistic regression model for prediction of CAHD

Categorical Variables	Univariate analysis OR (95% CI)	*P* value	Multivariate analysis OR (95% CI)	*P* value
Sex	0.507 (0.408–0.630)	< 0.001	1.947 (1.353–2.801)	< 0.001
Age	0.654 (0.527–0.811)	< 0.001	0.961 (0.946–0.975)	< 0.001
Smokers	0.616 (0.489–0.776)	< 0.001	1.042 (0.700–0.951)	0.013
DM	0.542 (0.420–0.698)	< 0.001	1.641 (1.158–2.326)	0.005
Hypertension	0.543 (0.434–0.681)	< 0.001	0.649 (0.505–0.834)	0.001
BMI	0.927 (0.747–1.149)	0.489	0.950 (0.918–0.986)	0.416
LDL/HDL	0.347 (0.278–0.433)	< 0.001	0.473 (0.399–0.561)	< 0.001
LDL-C	0.694 (0.559–0.862)	0.001	0.746 (0.482–1.173)	0.209
HDL-C	2.178(1.748–2.715)	< 0.001	1.549 (0.625–3.827)	0.339
TC	0.797 (0.539–1.072)	0.134	0.980 (0.792–1.226)	0.882
CRP	1.026 (1.010–1.043)	0.001	0.960 (0.930–0.988)	0.034
HDU	0.915 (0.696–1.204)	0.527	1.153 (0.845–1.567)	0.370

*CAHD* coronary atherosclerotic heart disease, *LDL-C* low-density-lipoprotein cholesterol, *HDL-C* high-density-lipoprotein cholesterol, *LDL/HDL* the ratio of low-density-lipoprotein to high-density-lipoprotein, *DM* diabetes mellitus, *TC* total cholesterol, *TG* triglyceride, *BMI* body mass index, *OR* indicates odds ratio, *CI* confidence interval, *CRP* C-creactive protein, *HDU* Hypolipidemic Durg Use

**Table 9:** Multicollinearity of age, sex, hypertension, diabetes, smoking and LDL/HDL for CAHD

Categorical variables	Unstandardized coefficients beta	Coefficients std. error	Standardized coefficients beta	*t* Value	*P* Value	R	R^2^	△R
Constant	− 0.667	0.114		− 5.863	< 0.001	0.408	0.167	0.163
Sex	0.134	0.031	0.134	4.331	< 0.001			
Age	0.009	0.001	0.196	7.504	< 0.001			
Smokers	0.042	0.034	0.039	1.261	0.028			
DM	0.092	0.030	0.078	3.069	0.002			
Hypertension	− 0.090	0.026	− 0.088	− 3.418	0.001			
LDL/HDL	0.148	0.013	0.285	11.272	< 0.001			

*CAHD* coronary atherosclerotic heart disease, *LDL/HDL* the ratio of low-density-lipoprotein to high-density-lipoprotein

## Discussion

The occurrence and development of coronary atherosclerosis result from multiple factors. Dyslipidemia, as a known risk factor, promotes and aggravates coronary atherosclerosis. Increasing evidence shows that the decrease of HDL-C and the increase of LDL-C may be involved in the progression of atherosclerosis and promote the development of CAHD [18–20]. Elevated LDL-C level is an independent predictor of atherosclerotic cardiovascular disease. Decreased LDL-C level can reduce the incidence of cardiovascular disease and cardiovascular events such as AMI and ischemic stroke [21]. In contrast to LDL-C, increased HDL-C level can effectively slow down atherosclerosis and reduce the occurrence of atherosclerosis-related diseases [22]. Consistent with these results, our study shows the level of LDL-C is higher and the level of HDL-C is lower in CAHD group than those in non-CAHD group.

A number of clinical studies have shown that in addition to single blood lipids, elevated LDL/HDL is a risk factor for coronary atherosclerosis [23, 24]. In accordance with the results of previous studies, observation on 1351 participants, shows that the LDL/HDL ratio in CAHD group was higher than that in non-CAHD group. To understand the value of LDL/HDL indicators, we further explored the boundary-value of LDL/HDL ratio according to the severity of coronary atherosclerosis using the Gensini score system. The results suggested that a progressive increase in the ratio of LDL/HDL was present for individuals with an increasing degree of coronary vascular stenosis and a higher Gensini scores. Compared with a single blood lipid index, the LDL/HDL ratio can detect the earlier imbalance between atherosclerotic and anti-atherosclerotic lipoproteins, and can effectively reflect the coronary atherosclerotic severity.

Our study shows that LDL/HDL ratio which combining the two variables is better than either LDL-C or HDL-C alone in predicting coronary atherosclerotic severity, as an independent risk factor for CAHD. According to the ROC curve analysis, the cut-off value of LDL/HDL ratio was 2.517, and the sensitivity and specificity for prediction of CAHD are 64.5% and 61.3%, respectively. As expected, LDL/HDL ratio was predictive for CAHD both in univariate and multivariate analyses. But LDL-C and HDL-C were predictive for CAHD only in univariate analysis. All these data suggested LDL/HDL ratio is better than LDL-C or HDL-C as an independent predictor for CAHD. LDL/HDL ratio is considered to be a sensitive predictor of CAHD, especially if the values are ≥ 2.517. It has an important clinical significance for prevention of CAHD and guiding therapy of coronary atherosclerosis. Using this cutoff, we can identify early high-risk CAHD patients and make effective interventions to reduce LDL/HDL ratio, thereby reducing the incidence of CAHD. Consequently, we used truncation criteria to verify the risk factors for CAHD and demonstrated that the incidence of CAHD increased and the degree of coronary artery stenosis significantly increased in the group with LDL/HDL ratio over 2.517.

Blood lipid level also depends on age, smoking, diabetes, hypertension and drugs. We investigated LDL/HDL in different conditions and found it is not affected by diabetes mellitus, hypertension, and smoking status in multivariate analyses. Patients with AMI were not included in the study because acute ischemic events may affect blood lipid level. Age is the risk factor of diabetes mellitus, hypertension, smoking status. The proportion of lipid drug treatment before admission was low and not different between CHAD and non-CHAD group. Diabetes is an important risk factor for CAHD and is closely related to dyslipidemia. There was no difference of LDL/HDL between DM and non-DM group, but in all participants LDL/HDL had a positive correlation with the level of fasting blood glucose using Pearson correlation analysis. Since, it has a series of confounding factors that affect the analysis and interpretation of the results. This contradict result is worth further study.

Our study predicts the value of LDL/HDL for CAHD independent of the factors of age, sex, hypertension, diabetes, smoke. We have separately calculated LDL/HDL and CAHD risk factors using the multicollinearity regression model. The result showed that VIF is near to 1, which suggests there is no multicollinearity between LDL/HDL and age, sex, hypertension, diabetes mellitus, smoking status. However, there is the collinearity among LDL-C, HDL-C and LDL/HDL (the data is not attached). LDL/HDL is calculated from LDL-C and HDL-C, so multicollinearity is inevitable.

## Limitations

Admittedly, our studies have some limitations. (1) This study was limited by its retrospective design and single-center nature. Selection bias may also be present in baseline characteristics, so the findings may not be applicable to the general population. (2) It is a cross-sectional study with many interference factors, the confounding biasis hard to exclude in statistical analysis. (3) We did not conduct a follow-up studies; Effect of the LDL /HDL ratio on the prognosis of patients with CAHD needs further study. The limits mentioned above also indicate the promising potential of this study, which lays the foundation for our future studies.

## Conclusion

To summarize, LDL/HDL ratio played an essential role in CAHD, acting as a representative of CAHD severity. LDL/HDL ratio has higher specificity and sensitivity than the single indicator LDL-C or HDL-C. Adding LDL/HDL to traditional risk factors can further improve the comprehensive judgment index for the occurrence of coronary atherosclerosis. LDL/HDL ratio may identify early patients with atherosclerosis or high-risk individuals for CAHD, guiding primary prevention strategies for CAHD.

## Data Availability

The datasets generated and analyzed during the current study are available from the corresponding author on reasonable request.
